# A Contemporary Study of Pre-hospital Traumatic Cardiac Arrest: Distinguishing Exsanguination From Non-exsanguination Arrest With a Review of Current Literature

**DOI:** 10.7759/cureus.48181

**Published:** 2023-11-02

**Authors:** Eduardo Serpa, Steve O Zimmerman, Zachary M Bauman, Narong Kulvatunyou

**Affiliations:** 1 Surgery, Central Michigan University College of Medicine, Saginaw, USA; 2 Acute Care Surgery, University of Arizona College of Medicine-Tucson, Tucson, USA; 3 Surgery, University of Nebraska Medical Center, Omaha, USA

**Keywords:** ­trauma, survival, exsanguination, pre-hospital, traumatic cardiac arrest

## Abstract

Background

Traumatic cardiac arrest (TCA) remains a challenging problem in terms of diagnosis and management. This is due to difficulty distinguishing the TCA cause and therefore understanding the pathophysiology. The goal of this study was to analyze a contemporary series of TCA patients and classify the causes of TCA into exsanguination (EX) arrest and non-exsanguination (non-EX) arrest.

Methods

This was a retrospective review of patients suffering TCA during 2019 at a level I trauma center. We excluded patients whose arrests were from medical causes such as ventricular fibrillation, ventricular tachycardia, pulmonary embolus, etc., hanging, drowning, thermal injury, outside transfer, and pediatric patients (age <13 as this is our institutional definition for pediatric trauma patients). We reviewed pre-hospital run-sheets, hospital charts including autopsy findings, and classified patients into EX and non-EX TCA. We defined a witnessed arrest (WA) using the traditional outside hospital cardiac (non-trauma) arrest definition. Outcomes included the incidence of EX arrest, survival to discharge, and hospital costs. Descriptive statistics were used. Significance was set at p < 0.05.

Results

After exclusion, 54 patients suffered TCA with a mean age of 45.9 (±19.8) years. Eighty-three percent of patients were male. The average cost per TCA was ~$16,000. Of the 54 TCAs, 26 (48%) were WA, with one (1.85%) survivor (no non-WA TCA patients survived). Twenty-two (41%) patients died from EX-arrest; 59% penetrating vs. 28% blunt (p = 0.03). The one EX-arrest survivor was a 19-year-old gunshot wound to the leg whose arrest was witnessed, with a short downtime, and the cause of arrest (bleeding leg wound) was quickly reversible.

Conclusion

We classified 41% of TCAs to have died from EX-arrest with only a 1.85% survival rate. This study calls for a TCA pre-hospital registry with accurate and consistent data definitions and collection. The registry should capture the cause of arrest for future research, management decision-making, and prognostication.

## Introduction

Traumatic cardiac arrest (TCA) remains a clinician’s enigma in terms of management and prognostication, starting from the pre-hospital setting, progressing through the emergency department, and continuing through the hospital course. Existing outcome and prognostication reports vary widely within the literature [1,2], which are due to the differences in the mechanism of injury, the difference in pre-hospital management, which varies geographically, the difference in pre-hospital time, and differences with in-patient management [3,4]. One of the difficulties in gaining insight into TCAs in all published series is the unknown or unconfirmed cause of each TCA, which sets TCA apart from its counterpart and widely published non-TCA arrest (an arrest due to a cardiac origin), with which the cause is often ventricular fibrillation or ventricular tachycardia (VF/VT) [5]. Despite not always knowing the underlying cause of the TCA, pre-hospital medical personnel are required to follow and adhere to resuscitation guidelines according to non-TCA recommendations [6,7], which often consist of external cardiac compression and/or administration of intravenous epinephrine, etc. This resuscitation approach may or may not be beneficial to TCA patients, especially to those who suffered an exsanguination (EX) TCA. Without more research and understanding for the causes of TCA and the pathophysiology associated with each cause, one can never gain insight into TCA to provide the optimal management strategy. The goal of this study was to analyze a contemporary series of TCAs over a short period of time at an urban, level 1 trauma center. The objective of this study is to classify the causes of TCA into EX arrest versus non-EX arrest and highlight this distinction as a necessary component to direct future research. This will in turn allow for a better understanding of TCA pathophysiology, resuscitation and management of TCA, and prognostication after TCA.

## Materials and methods

This was a retrospective cohort study. We followed Strengthening the Reporting of Observational Studies in Epidemiology Reporting Guidelines [8]. The participants included trauma patients who suffered pre-hospital TCA in 2019. The geographic population included the city of Tucson, Arizona, and its surrounding area, which is an urban city with over 1.4 million residents. The city is served by two main emergency medical service (EMS) systems. The Institution Review Board of the University of Arizona approved this study.

Patients who qualified for the study were identified from the trauma registry. We excluded patients whose arrests were from medical causes such as ventricular fibrillation, ventricular tachycardia, pulmonary embolus, etc., hanging, drowning, thermal injury, outside transfer, and pediatric patients (age <13). At our institution, pediatric trauma patients are defined as those under the age of 13 years.

For data collection, we reviewed the EMS run-sheet report to obtain EMS pre-hospital information, which included dispatch and response time (time from the call center to the time of arrival at the patient), scene time, transport time, total pre-hospital time, and any interventions performed in the pre-hospital setting including airway management (endotracheal intubation versus King airway versus no intubation), intravenous access versus intraosseous access, if cardiopulmonary resuscitation (CPR) was performed and by whom (classified as bystander, by EMS at scene, or by EMS in route) and for how long, and if epinephrine was administered (total amount given). A King airway is used by pre-hospital providers to secure the airway in the field. It is similar to an endotracheal tube but is placed without laryngoscopy with a balloon in the esophagus and a second balloon in the hypopharynx. The balloon in the esophagus occludes the esophagus, while the balloon in the hypopharynx is where the patient is ventilated and oxygenated. For this study, the authors specifically defined patients having a witnessed arrest (WA) as someone who was witnessed by EMS personnel to have signs of life (patient was awake, alert, spontaneously breathing, and/or spontaneously moving), then becoming unconscious and unresponsive, stop breathing or moving, and had no pulse, regardless if an electrocardiograph (EKG) was obtained or recorded. We mirrored this WA definition after a non-TCA outside-of-hospital cardiac arrest (OHCA). When reviewing the TCA literature as well as our current information from our local pre-hospital practice, the authors could not always be certain of the exact moment an arrest began, as it was often not well documented. Upon detailed review of the pre-hospital records, the arrest could have begun at one of the following time points: loss of consciousness, cessation of breathing, or loss of pulse with organized electrical activity (pulseless electrical activity, PEA) or without organized activity (VF/VT or asystole) [9]. 

We further reviewed the electronic medical record once the patient arrived at our hospital and obtained data from the trauma nurse flowsheet, emergency department (ED) physician notes (part of the trauma resuscitation team but with a different perspective), trauma history and physical exam, operative notes, and hospital course and outcomes. The authors then reviewed the autopsy findings and determined the most likely cause of death, classifying it into either an EX-arrest or a non-EX arrest. EX-arrest was based on clinical information of significant blood loss at the scene, in the ED, in the operating room (OR), or autopsy findings of a significant amount of blood (more than 1.5 liters) found in the thorax, abdominal, or retroperitoneal cavity along with named organ injuries, as well as blood loss associated with long bone fractures. If no significant amount of blood loss was identified anywhere, the cause of arrest defaulted to a non-EX arrest, which could be from hypoxia, cervical spinal cord injury (supported by autopsy findings), possible tension pneumothorax, possible blunt cardiac pump failure from cardiac contusion, or cardiac tamponade if there was blood surrounding the heart in an enclosed pericardial sac.

Our primary outcome for the study was the incidence of EX-arrest. Secondary outcomes included survival to hospital discharge, survival to organ donation [10,11], and hospital costs. All data was entered into the Microsoft Excel 2019 spreadsheet (Microsoft Corp., Redmond, WA). Statistical calculations were performed using STATA 14 (College Station, TX). We used descriptive statistics to describe continuous variables as a mean plus standard deviation (STD) or median (interquartile range (IR)), and category variables as a percentage or proportion. Statistical significance was set at a p-value < 0.05.

## Results

**Figure 1:**
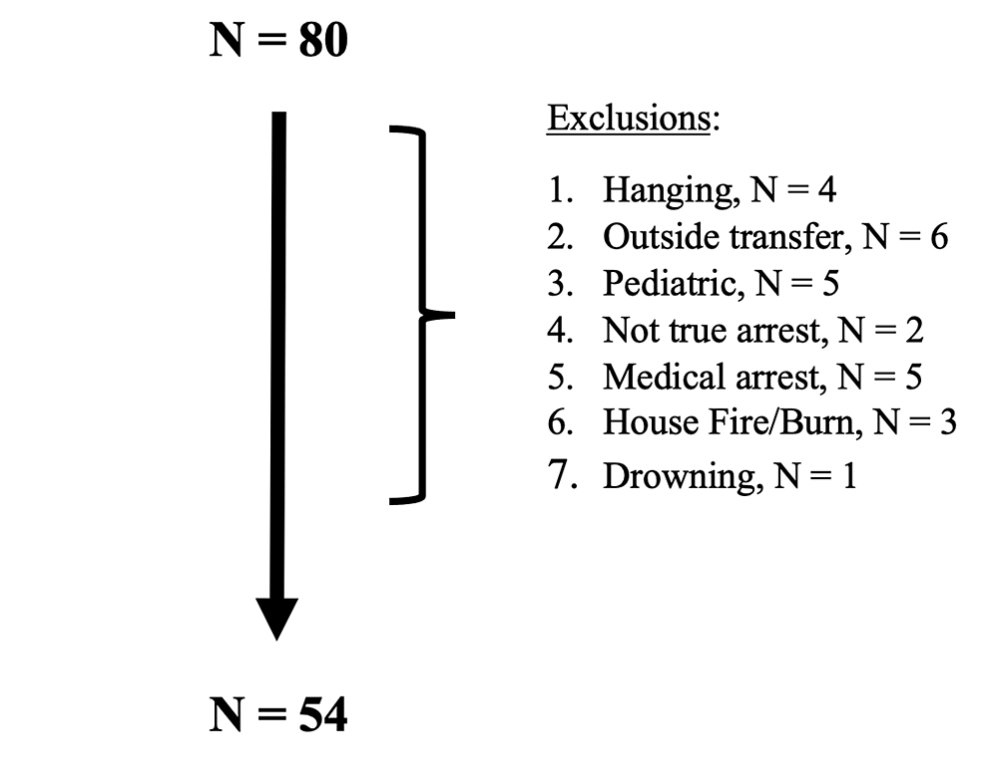
Patients who suffered traumatic cardiac arrest (TCA).

After the exclusion, there were 54 patients who suffered TCA in this series (Figure 1). Table 1 summarizes our overall findings. The population’s mean age was 45.9 (±19.8) years, 83% were male, and 59% were from blunt traumatic cardiac arrest. The average pre-hospital time for this study and setting was 30 (±10) minutes, which appeared relatively long for this group of trauma patients. We found 48% of our TCA patients had a WA according to our definition mirroring the OHCA definition, which means a little more than half of our TCA patients had an unwitnessed arrest with presumed additional unknown downtime. The primary outcome (incidence of EX-arrest) was 41% and significantly higher in penetrating than in blunt trauma (59% vs. 28%, p = 0.03). The penetrating TCA patients who did not die from exsanguination all died from the sequela of a gunshot wound (GSW) to the head (N = 9). There was one survivor (described below), giving our contemporary series a survival rate of 1.85%. The average cost for reviving one TCA patient was $15,949.

**Table 1:** Population cohort summary. WA was defined in the manuscript as someone who was witnessed as awake/alert, spontaneously breathing and moving, then became unconscious and unresponsive, stopped breathing, with loss of pulse, regardless of electrocardiographic tracing when it was obtained or recorded. Auto vs. Ped: automobile struck pedestrian; CPR: cardiopulmonary resuscitation; EMS: emergency medical service; EX: exsanguination arrest; GSW: gunshot wound; IR: interquartile range; MVC: motor vehicle collision; MCC: motorcycle collision; non-EX: non-exsanguination arrest; SW: stabbed wound; TCA: traumatic cardiac arrest; SD: standard deviation.

Characteristic	Value
Total patients suffering TCA, n	54
Age, mean (SD)	45.9 (19.8)
Gender, male (%)	83
Mechanism of injury, blunt (%)	59
Penetrating ratio, GSW: SW	21: 1
Blunt mechanism breakdown	
MVC, n	13
MCC, n	8
Auto vs Ped, n	9
Bicyclist, n	1
Hit by train, n	1
Time	
Dispatch/response, minutes (SD)	7.2 (2.6)
Scene, minutes (SD)	10.4 (5.3)
Transport, minutes (SD)	13 (5.8)
Total, minutes (SD)	30 (9.5)
Witnessed arrest (WA)* by EMS, %	48
CPR	
Bystander, n	14
EMS at the scene, n	16
EMS in route, n	21
Unknown, n	3
EX vs non-EX arrest ratio (% EX)	22:32 (41)
Blunt, n = 32 (% EX)	9:23 (28)
Penetrating, n = 22 (% EX)	13:9 (59)
Survived, n	1
Cost, $, median (IR)	15,949 (14,213; 22,130)

In Table 2, the authors grouped TCA patients into WA (N = 26) versus non-WA (N = 24) and correlated them with the initial EKG and timeline. There were, however, nine patients (17%) who had missing initial EKGs. It was interesting to note that even among the WA, the initial rhythms encountered varied from an organized sinus rhythm to an asystole. The findings were unable to determine if there was any relationship between the initial EKG and the cause of arrest. One WA patient survived, while none of the non-WA TCA survived. The one WA arrest who survived had the initial cardiac rhythm of sinus bradycardia. His case is being described below to provide further insight into this scenario. It represented the best-case scenario of a pre-hospital EX-arrest in which the arrest was witnessed, the downtime and the transport time were short and precisely captured (five minutes), and the source of the exsanguination (leg wound) was known and easily controlled and repaired. The patient had a good outcome clinically and neurologically. 

**Table 2:** Witnessed arrest versus non-witnessed arrest according to our definition.

	Initial rhythm	Dispatch time (min)	Scene time (min)	Transport time (min)	Total time (min)
Witnessed arrest, N = 26	Asystole, N = 4	8 ± 2.6	9.8 ± 4	17.8 ± 6	33.8 ± 11.5
	PEA, N = 3	9.7 ± 2.1	13 ± 12	12.7 ± 8.6	35 ± 13
	Sinus, N = 13	7.8 ± 2.9	8 ± 2.8	12 ± 4.8	28 ± 7
	No recorded EKG rhythm, N = 6				
Unwitnessed arrest, N = 24	Asystole, N = 7	6 ± 1.7	8.6 ± 3.5	11 ± 3.6	26 ± 7.4
	PEA, N = 11	6.5 ± 2.4	12 ± 5.7	11.2 ± 4.3	29.6 ± 8.3
	V-Fib, N = 2	6.5 ± 3.5	17 ± 5	13 ± 4.2	37 ± 12.8
	Sinus, N = 1	5	16	31	52
	No recorded EKG rhythm, N = 3				

Case review of witnessed TCA with survival

A 19-year-old male suffered a GSW to his left thigh just above the knee. The first responders (police) had already placed a tourniquet above the wound as soon as they arrived on the scene. When EMS arrived, the patient was not responding but was breathing and moaning. When the patient was loaded into the ambulance, his breathing and moaning stopped (WA by EMS personnel). Cardiac compressions ensued, and three rounds of epinephrine were given. This WA and the downtime before arrival to the trauma bay was approximately five minutes. In the emergency department, the patient was noted to be in asystole with fixed and dilated pupils. He underwent immediate left-sided resuscitative thoracotomy (RT) in the ED trauma bay, and the aorta was cross-clamped. He received four units of packed red blood cells and three units of fresh frozen plasma with cardiac massage and a single intracardiac epinephrine injection with the return of spontaneous circulation. He was immediately taken to the operating room for repair of his left above-the-knee popliteal artery injury and closure of his left RT. He survived and was discharged from the hospital four days after surgery, completely neurologically intact.

## Discussion

In this contemporary, albeit small, TCA series, we found 41% of TCA died from EX-arrest, one patient survived (1.85%), and there is an average cost of ~$16,000 per one resuscitation. Most trauma surgeons and TCA researchers understand that there could be various possible causes of TCA, but none of the prior series (Table 3) [4,5,12-40] specifically were able to group or classify the cause because most series were retrospective in nature over a long time period. Furthermore, most previous studies did not obtain a confirmatory autopsy study as well. Obtaining autopsy findings retrospectively is not always possible, and sometimes the information may be incomplete. But at our institution, beginning in 2019, our trauma surgeons have been vigilant to alert the pathologist of these TCA cases, and the senior author (NK) has been prospectively collecting and gathering the data in real-time. Only one recent study by Callcut et al., who published a multi-center study of the cause of trauma-related death, reported that 44.7% of their 546 pre-hospital cardiac arrests were from EX-arrest. However, in their study, 22% did not have a confirmatory coroner’s report [41]. This was very similar to our study, which demonstrated a 41% TCA mortality from EX-arrest. 

**Table 3:** Published series and survival of traumatic cardiac arrest. *Overlapping database; ^Percent of patients from that series that survived the trauma cardiac arrest from that mechanism of injury; ROC: resuscitation outcomes consortium; PROPHET: prospective observational pre-hospital and hospital registry for trauma.

Authors	Study period	Country	N	Blunt (%)^	Penetrating (%)^	Survived, N (%)
Shimazu et al. [12]	1976-1981	USA	267			7 (2.6)
Aprahamian et al. [13]	1981-1982	USA	95			3 (3.2)
Esposito et al. [14]	1985-1989	USA	112			2 (1.8)
Rosemary et al. [15]	1989-1991	USA	138	96	42	0 (0)
Stratton et al. [16]	1994-1994	USA	879	382 (1.3)	497 (0.8)	9 (1)
Battistella et al. [17]	1991-1996	USA	604	304	300	16 (2.6)
Martin et al. [18]	1997-2001	USA	110	110		1 (0.9)
Stockinger et al. [19]	1997-2002	USA	588			22 (3.7)
Pickens et al. [20]	1994-2001	USA	184			14 (7.6)
Willis et al. [21]	2001-2004	Australia	89			4 (4.5)
Tarmey et al. [22]*	2009-2010	Afghanistan War	52			4 (8)
Moriwaki et al. [23]	10-year	Japan	477			14 (3)
Molgerg et al. [24]	2003-2010	USA	294			1 (0.3)
Cera et al. [25]	1995-1998	USA	161			15 (9)
Alanezi et al. [26]	1992-2002	Canada	48			0 (0)
Lockey et al. [27]	10-year	England	871			68 (7.5)
David et al. [28]	1994-1996	Belgium/France	268			6 (2)
Huber-Wagner et al. [29]	1993-2004	Austria/Germany/Switzerland	757			136 (17)
Grasner et al. [30]	1998-2010	Germany	368			26 (7)
Deasy et al. [31]	2000-2009	Australia	545			15 (2.7)
Evans et al. [4]	05-07, 10-11	ROC, PROPHET	2,300			145 (6.3)
Leis et al. [32]	2006-2009	Spain	167			11 (7)
Morrison et al. [33]*	2006-2011	Afghanistan War	65			14 (22)
Kleber et al. [34]	2007-2013	Germany	52			15 (29)
Barnard et al. [35]	2009-2015	England/Wales	705			53 (7.5)
Beck et al. [36]	2008-2014	Australia	660			24 (15)
Konesky et al. [37]	2010-2014	USA	124			9 (7)
Barnard et al. [5]	2015-2017	England	304			10 (3.8)
Djarv et al. [38]	1990-2016	Sweden	1,774			66 (3.7)
Irfan et al. [39]	2010-2015	Qatar	410			10 (2.4)
Bhoi et al. [40]	2008-2013	India	1,061			3 (0.3)

Classifying the cause of TCA, especially in a registry such as the National Trauma Database, is very important for future research and understanding of TCA. Unlike its counterpart of cardiac arrest from a cardiac origin [6,7], no one really knows for sure if the standard CPR and administration of epinephrine are truly beneficial in EX-arrest patients [42-45]. Epinephrine has only been shown to help jump-start cardiac contractility after a cardiac standstill from ventricular fibrillation or ventricular tachycardia (VFIB/VTACH) [46]. In an experimental EX-arrest animal study, Kirimli et al. demonstrated that epinephrine was only effective after the intravascular volume resulting in circulatory collapse had been corrected [47]. Otherwise, there have been a few animal and clinical studies suggesting CPR might even be harmful in EX-arrest patients [42,43]. Consequently, if nearly half of the TCA patients could be from an EX-arrest, our current management and approach to helping these patients may not be appropriate.

We reported a survival rate of 1.85%, which is relatively low compared to the existing TCA literature, which ranges between 0.3% and 29% survival (Table 3) [4,5,12-40]. The two largest series, Evans et al. (N = 2300), which was a part of the Resuscitation Outcomes Consortium (ROC) and Prospective Observational Pre-hospital and Hospital Registry for Trauma (PROPHET) registries, and Djarv et al. (N=1774), which was a large Swedish National database, reported overall survival rates of 6.3% and 3.7%, respectively [4,38]. These ranges and wide variation in reported mortality outcomes among published series, as well as what the authors have discovered during the performance of this research, is why TCA needs a large prospective standardized registry, similar to what had been created for OHCA in the form of CARES (Cardiac Arrest Registry to Enhance Survival) [48-51]. Within the CARES, the pre-hospital data definitions and collection have been catered specifically to cardiac origin OHCA and it is prospectively collected. The registry includes a clear definition of WA, which we have applied to our study patients. While OHCA initial rhythms are VF or VT, in most TCA research, the initial rhythm is often defaulted to a PEA (pulseless electrical activity) with our study demonstrating this to be the initial rhythm 31% of the time [6,7]. However, no one really provides the exact type and morphology of the PEA arrest and how it may or may not correlate with the cause of the arrest [9,18,52,53]. Therefore, the authors believe that for us to better understand the management and prognostication of TCA, we need to have either a local, state, or national standardized TCA registry, or TARES (Traumatic Arrest Registry to Enhance Survival), that will allow us to have extensive pre-hospital data that include clearly defined and consistent data definitions and collection, a consistent definition of WA information, an initial EKG recorded, and what type and morphology of PEA arrest is present. This registry, with data prospectively entered, will provide us with the tool for future research into the management and prognostication of TCA, similar to what had been accomplished with the OHCA registry. 

This study, like many previous TCA studies, shared similar limitations, which include the retrospective nature, the imprecise and inconsistent definitions of the pre-hospital data, and the lack of a time point when arrest began due to an inconsistent definition of WA. As stated previously, the creation of a prospective registry would likely correct many of these issues. The authors did attempt to demonstrate consistent time points when the arrest occurred by using the OHCA registry definition of WA. Furthermore, there was a lack of initial EKG rhythms available, and in several cases where there were EKGs available, they lacked information about the morphology of the rhythm, which may have helped guide the management of the TCA patient. The authors of this study did attempt to determine the cause of each arrest using both clinical and autopsy findings; however, this needs to be correlated to the patient in real-time in order to make meaningful management decisions. Future studies should focus on these details to create a more standardized approach to guide resuscitation efforts. 

## Conclusions

In conclusion, the authors found in this contemporary, small TCA series that 41% of our TCA patients experienced an EX-arrest with a 1.85% survival rate. The authors call for the establishment of a local, state, and/or national pre-hospital registry for TCA, or TARES, that contains precise pre-hospital data definitions and collection, including a definition of WA and the initial EKG, as well as the cause of arrest using both clinical data and information gained from autopsy studies. This registry will provide us with a wealth of data for future TCA research, allowing for better management and prognostication of these patients.
